# Recent Progress in Nanoscale Covalent Organic Frameworks for Cancer Diagnosis and Therapy

**DOI:** 10.1007/s40820-021-00696-2

**Published:** 2021-08-16

**Authors:** Shuncheng Yao, Zhirong Liu, Linlin Li

**Affiliations:** 1 Beijing Institute of Nanoenergy and Nanosystems, Chinese Academy of Sciences, Beijing, 101400 People’s Republic of China; 2 School of Nanoscience and Technology, University of Chinese Academy of Sciences, Beijing, 100049 People’s Republic of China

**Keywords:** Covalent organic frameworks, Nanomedicine, Drug delivery, Cancer diagnosis and therapy

## Abstract

Covalent organic frameworks (COFs) as a type of porous and crystalline covalent organic polymer are built up from covalently linked and periodically arranged organic molecules. Their precise assembly, well-defined coordination network, and tunable porosity endow COFs with diverse characteristics such as low density, high crystallinity, porous structure, and large specific-surface area, as well as versatile functions and active sites that can be tuned at molecular and atomic level. These unique properties make them excellent candidate materials for biomedical applications, such as drug delivery, diagnostic imaging, and disease therapy. To realize these functions, the components, dimensions, and guest molecule loading into COFs have a great influence on their performance in various applications. In this review, we first introduce the influence of dimensions, building blocks, and synthetic conditions on the chemical stability, pore structure, and chemical interaction with guest molecules of COFs. Next, the applications of COFs in cancer diagnosis and therapy are summarized. Finally, some challenges for COFs in cancer therapy are noted and the problems to be solved in the future are proposed.

## Highlights

- Recent progress in nanoscale covalent organic frameworks (COFs)-mediated nanomedicines for cancer diagnosis and therapy is comprehensively summarized in this review.
- Future perspectives and challenges regarding COFs-mediated nanomedicines for diagnosis and therapy are discussed, with particular emphasis on possible clinical translation.

## Graphical abstract

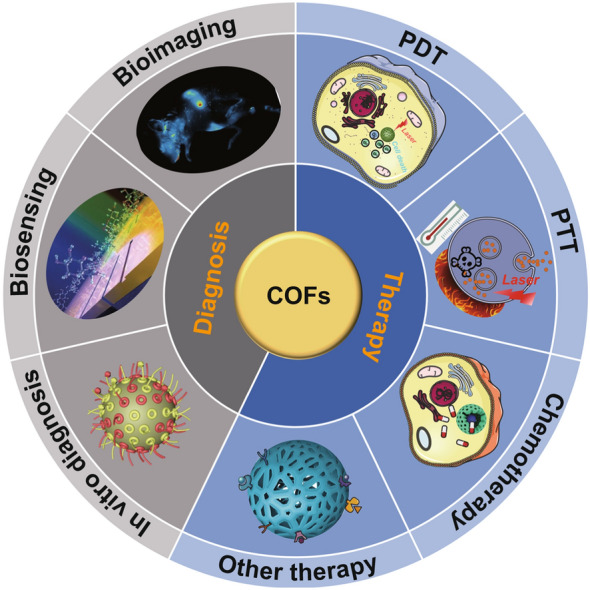

## Introduction

Porous nanomaterials, such as mesoporous silica nanomaterials, zeolites, and porous carbon nanomaterials, have showed potential applications in biomedical fields (including drug delivery, imaging, and biosensing) due to their large surface area, tunable porosity, and easy functionalization. However, these porous nanomaterials are often inert, lacking any chemical, biochemical, or physicochemical activity. In most cases, these chemically inert porous nanomaterials are useful only as carriers of other active species. More recently, however, with the development of “chemistry of the framework” [1], metal-organic frameworks (MOFs), and COFs have attracted great research interest in the fields of cancer diagnosis and therapy [2–4]. MOFs and COFs constructed from molecular building blocks through metal-ligand coordination bonds or covalent bonds can form periodic, programmed, and extended frameworks with extraordinarily high surface area, topological diversity, and aesthetically pleasing structures [5]. The major drawback of MOFs is their low stability, which may bring toxicity concerns and poor therapeutic response, thus restricting their biomedical applications [6].

To compensate for these limitations, a library of COFs have been designed and fabricated as alternatives to MOFs. In 2005, Yaghi et al. designed and synthesized the first COF [7]. COFs are crystalline porous organic polymeric frameworks mainly composed of light elements (H, B, C, N, and O) with periodical arrangements. They are built from molecular building blocks connected by covalent bonds, and thereby possess higher stability than MOFs. Over the last decade, researchers have continuously explored different types of linkers, building blocks, and reaction conditions to produce COFs with different properties. Structurally well‐defined COFs show impressive properties, including high specific surface area, precisely tailored pores and geometry [8–11], tailored and fully exposed active sites, flexible molecular structures [12–15], and low density [16], as well as unique electrical, magnetic, and optical characteristics. Given these properties, COFs with different structures and dimensions have been explored for gas storage and separation [17–19], heterogeneous catalysis [20–22], optoelectronics [23–26], and other applications.

**Fig. 1:**
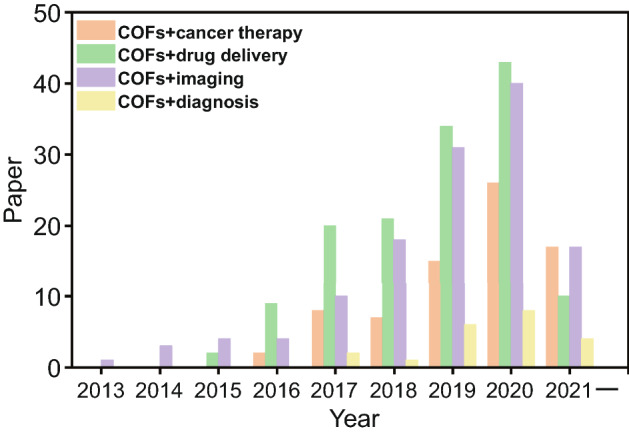
Paper on the ISI web of science with different themes between COFs and cancer therapy, drug delivery, imaging and diagnosis

Importantly, as a result of continuous research into molecular building blocks, surface functionalization, and fabrication methods, the structure and size of COFs can be continuously adjusted. Moreover, the size of COF can be reduced to nanoscale. Nanoscale COFs possess not only the good crystallization and porous characteristics of traditional COFs, but also the advantages of good dispersion, small volume, and high bioavailability. At the same time, their unique and tailorable nanostructures and properties make COFs ideal candidates for biomedical applications [27, 28], endowing them with high drug loading [29–33], outstanding therapeutic outcome [34–36], and sensitive sensing performance [37–39]. Although recent years, researchers have seen explosive growth in research on COFs for biomedical applications (Fig. 1), there is still a lack of comprehensive review papers to summarize the influence of COFs’ characteristics on their application performance. In this review, we focus on the fabrication optimization of two-dimensional (2D) and three-dimensional (3D) COFs and their applications for cancer diagnosis and therapy. First, we discuss the optimization of different performances of COFs and summarize the influence of their dimensions, building blocks, and synthetic conditions on their characteristics, including chemical stability, pore structure, and chemical interaction with guest molecules. Next, we summarize the application of COFs in cancer diagnosis and therapy. Finally, some problems and future perspectives of COFs in cancer diagnosis and therapy are discussed.

## Performance Optimization of 2D/3D COFs

The structures and properties of COFs are determined by the reactive and functional groups of their building blocks, which have been discussed in detail in some other reviews [40–43]. Typically, COFs are synthesized through reactions between different functional groups, and some widely used building blocks and linkages for COFs are listed in Table 1. So far, the covalent bond connecting groups mainly include –B–O–, –C–N–, –C–C–, and –C–O–. In this section, we discuss the influence of dimensions, building blocks, functional groups and reaction conditions on the properties of COFs, including their chemical stability and pore structure [44–49].

**Table 1:**
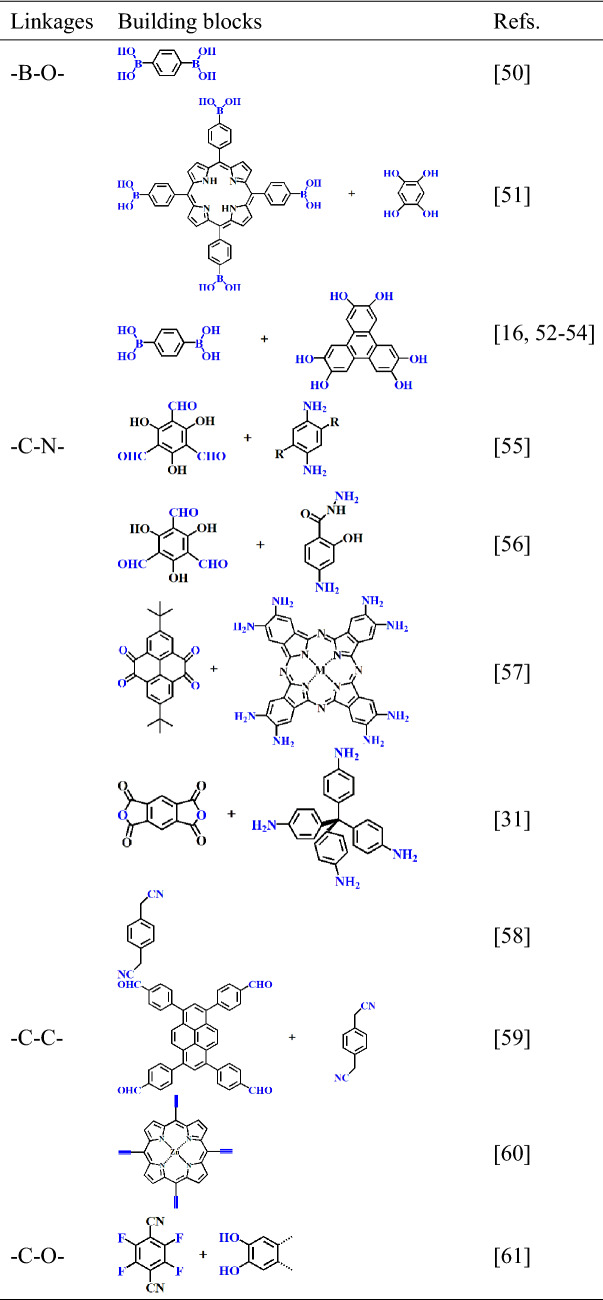
Main types of building blocks and linkages for fabrication of COFs

Since the discovery and rapid development of graphene, transition-metal dichalcogenides, graphitic carbon nitride (g-C_3_N_4_), 2D MOFs, and 2D COFs, 2D materials have been subject to extensive research attention. The “2D” in 2D COFs means that the covalent bonding is in the same plane; only the non-covalent interactions exist between the planes to form a 2D structure [62]. Compared with COFs of other dimensions, 2D COFs have the characteristics of ultrathin and relatively large surface area. 3D COFs have a three-dimensional expanded network, which are formed by nonplanar building blocks. Unlike 2D COFs, 3D COFs usually have more complex pore structures with features such as interpenetrated channels and cages, which are more conductive to separation, catalysis, guest molecule incorporation, and other functions. In addition, large surface area, low density, and abundant active sites are often accessible in 3D COFs.

Herein, we discuss the effects of dimensions, building blocks, functional groups and fabrication conditions on chemical stability [57, 63–69], pore structure [54, 70–72], and chemical interaction with guest molecules of 2D/3D COFs (Table 2).

**Table 2:** Factors affecting the performance of COFs

Properties	Influence factors	Refs.
Chemical stability	Building blocks	[73]
	Substituent group	[74]
Pore structure	Reaction media	[75]
	Reaction conditions	[75]
	Substituent group	[76]
Chemical interaction with guest molecules	Substituent group	[77]

### Chemical Stability

The building blocks and substituent groups of COFs are important parameters influencing their chemical stability. In recent years, the poor hydrolytic stability of COFs has become one of the biggest problems that plague their application. Researchers have found that the steric hindrance and instability of the unreacted groups, along with the skeleton structures formed by different building blocks, affect the stability of COFs. Stability can be effectively improved by changing the parameters of the framework, such as its building blocks and substituent groups [78].

**Fig. 2:**
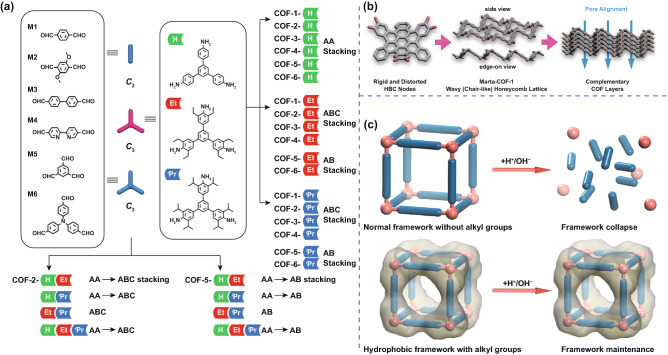
COFs with different chemical stability **a** Construction of the binary, ternary, and quaternary COFs and their stacking modes. Adapted with permission [81]. Copyright © 2018, American Chemical Society. **b** Schematic representation of the rigid and distorted HBC nodes and their incorporation in boronate ester COFs to obtain Marta-COF-1 with a wavy and highly complementary structure that guided the stacking of COF layers. Adapted with permission [79]. Copyright © 2019, American Chemical Society. **c** Normal framework without alkyl groups, in which the structure would collapse after being exposed to harsh environments, and hydrophobic framework with alkyl groups, in which the architecture could withstand harsh environments due to the protection of hydrophobic groups. Adapted with permission [74]. Copyright © 2020 Wiley‐VCH Verlag GmbH & Co. KGaA, Weinheim

For instance, Martínez-Abadía et al. [79] used polycyclic aromatic hydrocarbons as rigid nodes to form a 2D COF with a wavy honeycomb (chair-like) lattice (Fig. 2b). This wavy organic framework had high stability without damage to its charge transport characteristics. Li et al. [80] prepared a highly crystalline 2D COF of acylhydrazone by selecting building blocks with bond dipole moments; the spatial orientation of this framework proved conducive to the anti-parallel stacking. This method is widely applicable to hydrazide linkers containing various side-chain functional groups and surface activities. Using this strategy, COFs with high crystallinity and high yield of 1.4 g could be synthesized in a one-pot reaction within 30 min. The formation of high-crystallinity COFs was mainly attributed to the presence of the hydrazide linker and the formation of a large number of hydrogen bonds.

Xie et al. [73] synthesized a dibenzo[g,p]xanthogen COF (DBC-2P) with a large conjugated structure. Because the conjugated structure strengthened the interlayer interaction, the DBC-2P showed excellent stability in both strong acids and bases. In general, the polycyclic aromatic hydrocarbons, strong dipole moment, and large conjugated structure of the building blocks are conducive to the formation of a rigid structure with strong interaction between layers, thus improving the chemical stability of the COF.

Wu et al. [81] used the multivariable method to comprehensively control the layer stacking and chemical stability of a hexagonal network via managing interlayer steric hindrance (Fig. 2a). Specifically, a family of two-, three-, and four-component 2D COFs with AA, AB, or ABC stacking, respectively, was prepared by co-condensation of triamines both with and without alkyl substituents (ethyl and isopropyl) and a di- or trialdehyde. The alkyl groups were periodically appended on the channel walls and contents, which affected the crystal stacking energy and hydrolytic stability of the framework. The chemical stability of the 2D COFs was controlled by maximizing the total crystal stacking energy and protecting the hydrolytic-sensitive framework by kinetic blocking. The results showed that the COFs with a higher concentration of alkyl substituents had higher chemical stability. Ma et al. [74] further found that the stability of 3D COFs can be improved by periodically modifying the isopropyl groups on the backbone. More specifically, based on the Schiff-base chemistry, the condensation of tetraphenyl methane incorporating 2,6-diisopropyl aniline and terephthalaldehyde or 4,4′-biphenyldicarboxaldehyde resulted in two new varieties of 3D COFs. Due to the strong hydrophobicity of alkyl groups (Fig. 2c), the COFs had high crystallinity, permanent pores, and high stability in harsh environments such as strong acids (3 M HCl or 3 M H_2_SO_4_ for one week), strong alkalis (20 M NaOH for one week), and boiling water (100 ºC for one month). This work demonstrated the application of alkyl modification to adjusting chemical stability. These studies confirm that changing the substituent groups can affect the stability of a COF. The researchers found that increasing the alkyl substituent content both enhanced the crystal formation and hydrophobicity of the COF, and improved its hydrolytic stability [81].

### Pore Structure

It is expected that both 2D and 3D COFs have high loading of guest molecules and predictable release behavior due to their highly ordered pore structure, which makes them a new candidate for loading and release of drugs. The control of the microstructure and pore parameters of COFs has thus been a topic of intense attention to researchers. Such control remains a major challenge; however after removing the guest species located in the pores, structural interpenetration and pore collapse often occur. Recently, though, researchers have found that the reaction media, unreacted groups, and bonding types play important roles in the regulation of pore properties, including porosity and pore size. For instance, Feng et al. [75] reported that the morphology and pore parameters of 2D COFs can be controlled by adjusting the reaction time. Zinc(II) 5,10,15,20-tetrakis(4-(dihydroxyboryl)phenyl) porphyrin (TDHB-ZnP) was utilized in conjunction with 1,2,4,5-tetrahydroxybenzene (THB) as building units for the construction of a 2D COF. The surface area and pore volume of this COF increased with the prolongation of reaction time. After 15 days of reaction, the surface area had increased from 85 to 1742 m^2^ g^–1^ while the pore volume had increased from 0.069 to 1.1153 cm^3^ g^–1^, demonstrating that reaction conditions play an important role in regulation of pore properties. In particular, the extension of reaction time can improve the surface area and porosity of a COF.

At the same time, the pore properties of a COF can be well regulated by adjusting the reaction solvents. For example, Feng et al. [75] reported the first example of synthesis of two-dimensional porphyrin COF via boron esterification under solvothermal conditions, and comprehensively controlled its macrostructure and pore parameters in the production process. Specifically, zinc(II) 5,10,15,20-tetrakis (4-(dihydroxycarbonyl) phenyl) porphyrin (TDHB-ZnP) was utilized in conjunction with 1,2,4,5-tetrahydroxybenzene (THB) as building units for the construction of the 2D COF. A series of COFs with controllable porosity can be synthesized using the mixture of trimethylbenzene and dioxane in different ratios as the condensation solvent. Bi et al. [82] prepared a 2D COF using a condensation reaction of 3,5-dicyano-2,4,6-trimethylpyridine with linear/trigonal monomers on arylmethyl carbon atoms. The 2D COF was linked by trans-disubstituted C=C bonds via condensation at arylmethyl carbon atoms, which experienced the reversibility of the C=C bonds in a Knoevenagel condensation reaction, allowing the formation of a crystalline honeycomb structure with high porosity (1235 m^2^ g^–1^).

**Fig. 3:**
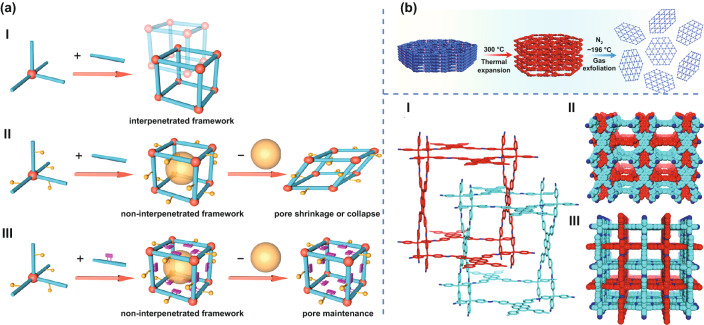
**a** COFs with different pore structures and chemical interaction with guest molecules I. Interpenetrated microporous framework constructed from undecorated edges. II. Non-interpenetrated mesoporous framework built up from partially decorated edges, in which the pore shrinkage or collapse would happen after the removal of guest molecules. III. Non-interpenetrated mesoporous framework created by highly decorated edges, in which the pores can be maintained entirely after the removal of guest molecules. Adapted with permission [76]. Copyright © 2020 American Chemical Society. **b** Schematic diagram for the temperature-swing gas exfoliation of NUS-30 from bulk layered powder to ultrathin 2D nanosheets. Adapted with permission [83]. Copyright © 2018 American Chemical Society. **c** I. Two-fold interpenetrated PTS network; II. space-filling model viewed from the A axis; III. space-filling model viewed from the C axis. Adapted with permission [77]. Copyright © 2016 American Chemical Society

Wang et al. [76] reported a series of 3D mesoporous COFs through steric hindrance engineering. Strategically introducing methoxy and methyl groups on the monomer produced a non-interpenetrating 3D diamond structure with permanent mesopores (up to 26.5 Å) and high specific surface area (> 3000 m^2^ g^–1^), which was superior to conventional COFs with the same topology (Fig. 3a). This work opens up a new way to create 3D mesoporous COFs, which have potential applications in the adsorption and separation of inorganic, organic, and biological molecules. Thus, not only the regulation of different substituents can affect the chemical stability of COF, but also the selection of rigid substituents can contribute to permanent mesopores and high specific surface area.

### Chemical Interaction with Guest Molecules

Due to their porous nature, COFs have attracted wide attention from researchers for applications including gas storage, sensing, and drug delivery. For drug delivery, the loading of drug molecules that can be regarded as guest molecules is determined by not only pore size and geometry, but also their chemical interaction with the functional groups of COFs. Researchers have found that changing the number and type of substituent groups in the COF structure can effectively regulate the interaction between COFs and specific guest molecules. Interestingly, in the process of design and synthesis of COF, the type and number of functional groups can be easily changed. For instance, Dong et al. [83] reported the synthesis of three kinds of azine-linked and imine-linked 2D COFs using monomers containing aggregation-induced emission (AIE) rotor-active tetraphenyl ethylene (TPE) moieties, and successfully prepared ultrathin 2D nanosheets (2−4 nm in thickness) using a temperature-swing gas exfoliation approach (Fig. 3b). The affinity between nanosheets and amino acids can be controlled by changing the number of azine groups, which can tune the key geometric interaction between the groups for a stronger host–guest combination by density functional calculations.

Compared with 3D COFs, 2D COFs have a wider space for interaction with guest molecules because of their ultrathin nature and large planar structure. That being said, 2D COFs can interact with guest molecules with larger molecular weight via π–π stacking of these molecules on their planar surface. Conversely, 3D COFs have some unique advantages in their interaction with guest molecules. For example, 3D COFs have high specific surface area and a large number of open sites, which can interact with more guest molecules. Gao et al. [84] have reported the design and synthesis of isostructural 3D COFs with different substituents. By changing the pore environment of the 3D COFs, the materials demonstrated selectivity to different guest molecules. They designed three isostructural 3D COFs with -H, -Me, or -F substituents, which had selectivity for CO_2_ over N_2_. These results have improved our understanding of the mesoporous environment of 3D COFs and their future applications. Based on the same principle, Lu et al. reported a general strategy for preparing 3D carboxyl COF by modifying hydroxyl COF after synthesis, and applied it for selective extraction of lanthanide ions [85]. The obtained COF had a good adsorption selectivity to Sr^2+^ and Fe^3+^. Lin et al. reported the synthesis of a new-type 3D pyrene-based carbon fiber (3D-Py-COF) that was started from tetrahedral and rectangular building blocks connected via [4 + 4] imine condensation reactions [77]. The synthesized COF adopted a double-layer interpenetrating PTS topology (Fig. 3c) and demonstrated selective adsorption of CO_2_ over N_2_. In general, COFs can be designed to have unique interactions with a variety of guest molecules due to their high porosity and pore distribution, special 2D/3D structures, and easy functional group modification.

Substituent groups, as one of the most important factors in regulating the interaction between COF and guest molecules, have been of vital interest to researchers. Specifically, by changing the number of substituents, the affinity between COF and guest molecules can be well regulated, and the selectivity of COFs to different molecules can be achieved by changing different kinds of substituent groups.

## COFs for Cancer Therapy

### Properties of COF

One danger associated with using traditional cytotoxic drugs in cancer therapy is that the drugs might accumulate in various tissues and organs in the body, causing serious side effects and inefficient therapeutic outcome. As a polymer network composed of repeating organic units, COFs have attractive features as drug delivery systems, including: (1) high surface area and large porosity, which can facilitate high drug loading and controlled drug release kinetics; (2) tunable size and high physiological stability to achieve effective tumor targeting and intratumoral accumulation; (3) easy surface functionalization of targeting ligands for targeted delivery of the drug molecules at desired locations; and (4) intrinsic multifunctionality for synergizing drug delivery with other therapeutic modalities, thus improving therapeutic efficacy. Because of their versatility and multifunctionality, COFs have largely bypassed the limitations of traditional anti-tumor drugs. Recently, COFs have been applied as carriers of drugs including doxorubicin (DOX) [29, 86], cisaconityl-doxorubicin (CAD) prodrug [87], pirfenidone (PFD) [88], ibuprofen (IBU) [31], and carboplatin [89] for disease treatment. Unlike other porous materials, COFs have rich π-conjugated structure and are easy to modify, which make them suitable for loading with a variety of drug molecules through hydrogen bonding, π–π stacking, host–guest interaction, and other methods. The above advantages might increase the loading capacity of drug molecules and broaden the modes of drug delivery.

In the process of phototherapy, light-absorbing organic dyes acting as photosensitizers (PSs) [35, 90–92] can absorb light and activate O_2_ in reactive oxygen species (ROS) that elicit cell oxidative damage and subsequent death. However, small-molecular PSs often suffer from poor water solubility, occasional photobleaching, high phototoxicity, low cell permeability, and nontargeted biodistribution, all of which result in insufficient ROS production and ineffective PDT. COFs with well-defined structures and compositions have recently shown considerable promise as potential PDT platforms [34, 90, 93, 94]. On the one hand, COFs are promising carriers for PSs due to the following characteristics: (1) the extended π-conjugation can improve the photoelectric performance and optimize the absorption band of the loaded PSs, thus improving the efficiency and penetration depth of PDT and optimizing the therapeutic effect via adjustment of the light-absorption range; (2) COFs can promote the diffusion of both molecular oxygen and the generated ROS [34], which can dynamically increase PDT efficiency; and (3) photosensitizers can be successfully nanometerized via the COF platform to avoid aggregation and self-quenching, so as to increase their endocytosis by the cells to optimize the PDT performance. On the other hand, the COFs formed with small molecular PSs and/or their derivatives as building blocks have excellent PDT effect, which is mainly due to the following reasons: (1) the large surface area of COFs can increase their light-harvesting ability [95]; and (2) the formation of COF structure can contribute to regulating the energy transfer pathway and exciton utilization, so as to improve the efficiency of ROS production [96].

In terms of photothermal conversion, COFs as an emerging class of organic crystalline porous material have good biocompatibility, high absorption cross section, high light-to-heat conversion efficiency with good photostability, and minimal dark toxicity, all of which make them promising candidates as PTAs. For PTT, COFs have many unique properties: (1) the π-conjugated structure of COFs can broaden and red-shift the absorption spectrum of the material, which can increase the penetration depth of light while also expanding the absorption band of light and improving the utilization of photons so as to enhance the therapeutic effect of PTT; (2) COF materials stacked together can produce a periodic π-array, which is conducive to the stability of electrons and the reduction of fluorescence quantum yield, and the resultant increased energy involved in the non-radiative transition enhances the PTT performance; and (3) given the versatile structure and functional groups of COFs, imaging agents can be integrated into COF, thereby realizing imaging-guided therapy.

### Applications of COFs

#### Chemotherapy

In cancer therapy, traditional chemotherapeutic drugs may accumulate in various tissues and organs of the body, resulting in serious side effects and inefficient treatment. In recent years, researchers have found that COF can act as an alternative drug delivery carrier to solve these problems, at least to an extent. In 2015, Yan et al. reported the first example of drug delivery using COF as carrier [31]. 3D porous crystalline polyimide COFs (PI-COFs) were synthesized by reacting pyromellitic dianhydride (PMDA) with tetrahedral building blocks of 1,3,5,7-tetraaminoadamantane (TAA) and tetra(4-aminophenyl) methane (TAPM), which had non- or interpenetrated structures. These 3D COFs showed high thermal stability and surface area (up to 2403 m^2^g^–1^), and 3D porous structure with 15 Å pores running along the *a* or *b* axis. Encouraged by the porous structure and high surface area of these frameworks, the authors chose IBU (molecular size of 5 × 10 Å^2^) as a model drug to study their drug loading and release capabilities. In tests, the PI-COFs showed high drug loading of 20 wt% with controlled drug release. The experimental results showed that most of the IBU was released after about 6 days, and the total delivery amount reached about 95% of the initial IBU loading. Zhang et al. packed anti-fibrotic pirfenidone (PFD) into an imine-based COF (COFTTA-DHTA), then modified it with poly(lactic-co-glycolic-acid)-poly (ethylene glycol) (PLGA-PEG) (abbreviated as PCPP) [88]. After intravenous injection, PCPP can accumulate and release PFD at the tumor site to downregulate the components of the tumor’s extracellular matrix (ECM), such as hyaluronic acid and collagen I. Such depletion of ECM largely decreased the solid stress of tumor and greatly alleviated its hypoxic state, thereby remodeling the ECM to enhance the efficacy of PDT, thus achieving a tumor suppression rate of 92%.

**Fig. 4:**
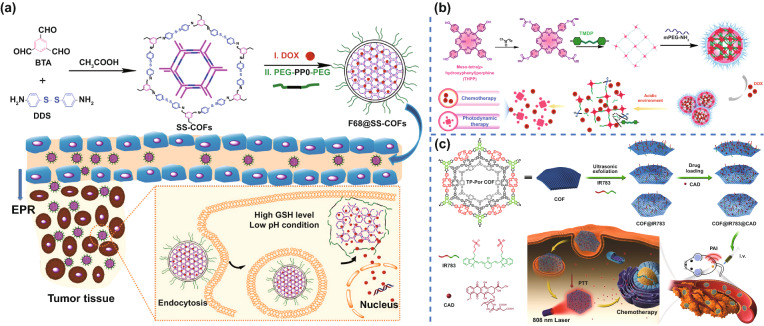
COFs for drug delivery. **a** Schematic illustration of the preparation of drug-loaded F68@SS-COFs and their intracellular GSH-responsive drug release. Adapted with permission [97]. Copyright © 2020 WILEY‐VCH Verlag GmbH & Co. KGaA, Weinheim. **b** Schematic illustration of the preparation of drug-loaded F68@SS-COFs and their intracellular GSH-responsive drug release. Adapted with permission [86]. Copyright © 2018 American Chemical Society. **c** Schematic illustration of the nanocomposite fabrication and cancer treatment process in vivo. Adapted with permission [87]. Copyright © 2019 American Chemical Society

In drug delivery, premature drug leakage usually leads to severe systemic toxicity and discounts drug release at the target site, resulting in multi-drug resistance and treatment failure. Therefore, there is an urgent need to develop drug carriers that have low drug leakage and can specifically release these drugs in tumor microenvironments (TME). Zhang et al. constructed redox-responsive 2D COF nanocarriers (denoted as F68@SSCOFs) for efficiently loading and delivering DOX [97]. These F68@SSCOFs were synthesized via the self-assembly of Pluronic F68 (an FDA-approved pharmaceutic adjuvant, PEG-PPO-PEG) and disulfide-containing COFs derived from a Schiff-base reaction between commercially available building blocks (4,4′-Diaminodiphenyl disulfide and 1,3,5-benzenetricarboxaldehyde). The obtained F68@SS-COFs had a large pore surface area up to 672 m^2^ g^–1^, π–π stacking interaction between COF and aromatic ring of DOX to realize a high drug loading, low premature leakage, and stimuli-responsive release in TME to kill tumor cells (Fig. 4a). The F68@SSCOFs rapidly released the loaded DOX in response to the glutathione (GSH) overproduced in tumors due to the redox-responsive involving disulfide bond [98]. Liu et al. prepared a pH-responsive covalent organic polymers (COP) (THPP-BAE-PEG COPs) [86]. The COPs, which contained pH-responsive cross-linked biodegradable β-amino esters (BAEs), were synthesized by the reaction between acryloyl meso-tetra(p-hydroxyphenyl) porphine (acryloyl-THPP) and 4,4′-trimethylene dipiperidine. Then, amine-modified poly (ethylene glycol) (PEG) was introduced to terminate the reaction and form the PEG shell (Fig. 4b). The resultant COP can be loaded with DOX, and has an extended blood circulation time (25 h) and effective anti-tumor effect after intravenous injection.

In addition to 3D COFs, 2D COFs have aroused wide attention due to their high dispersion, high accessible surfaces, and abundantly exposed active sites. Jia et al. synthesized a 2D COF (denoted as PEG-CCM@APTES-COF-1) via the self-assembly of polyethylene-glycol-modified curcumin derivatives (PEG-CCM) and amine-functionalized COF‑1 (APTES-COF-1) [29]. The 2D COF consisted of thin platelets with widths ranging from 120 to 150 nm. In vitro and in vivo studies showed that it had high DOX loading (9.71 wt%) and a high encapsulation efficiency of 90.5%, extended circulation time, and improved drug accumulation in tumors; these properties were mainly due to the surface modification of 2D COFs with PEG-CCM and the porous structure of 2D COF with improved drug loading.

In order to improve the circulatory and therapeutic effects of COFs, Chen et al. prepared a water-dispersible nanocomposite (COF@IR783), which was produced by the assembly of cyanine IR783 with COF [87]. The COF was prepared via the co-condensation reaction of 2,3,6,7,10,11-hexahydroxytriphenylene (HHTP) with 5,15-bis(4-boronophenyl)-porphyrin (Por), exhibiting a porous hexagonal framework with HHTP monomers at the angles and porphyrin monomers at the edges. Attributed to IR783, this 2D COF had nanoscale morphology (~ 200 nm) with good aqueous dispersibility and negative charge (− 36 mV), which were favorable for improving blood circulation time and enhancing the permeability and retention effect (EPR). In addition, the COF had good photothermal conversion performance, with photothermal conversion efficiency of approximately 15.5%. It was used as a drug carrier for prodrug cisaconityl-doxorubicin (CAD) (Fig. 4c). The combination of PTT with chemotherapy was found to significantly reduce the cell viability of 4T1 cells in vitro (the cell survival rate was 19.8%), and intravenous injection of COF@IR783@CAD into mice resulted in significant tumor ablation. Therefore, the reasonable material design can improve the circulatory and therapeutic effects of COFs.

Lalehan et al. fabricated a 2D imine-linked COF via the Schiff-base condensation reaction of building block 3,30-dimethoxybenzidine (DMB) and linking unit 1,3,5-triformylbenzene (TFB) under solvothermal conditions [89]. The resultant 2D COF had a high drug loading capacity for carboplatin (31.32%) with a zero-order, first-order, and Higuchi kinetic release behavior. The drug release from the 2D COF was minimum at the physiological pH of normal cells and fast at pH = 5.0, which was due to the weakening of the H-bonds between carboplatin and the COF in the acidic matrix. Therefore, this 2D COF demonstrates advantages over traditional polymer carriers, but its biological safety and in vivo toxicity need to be further studied.

#### Photodynamic Therapy

**Fig. 5:**
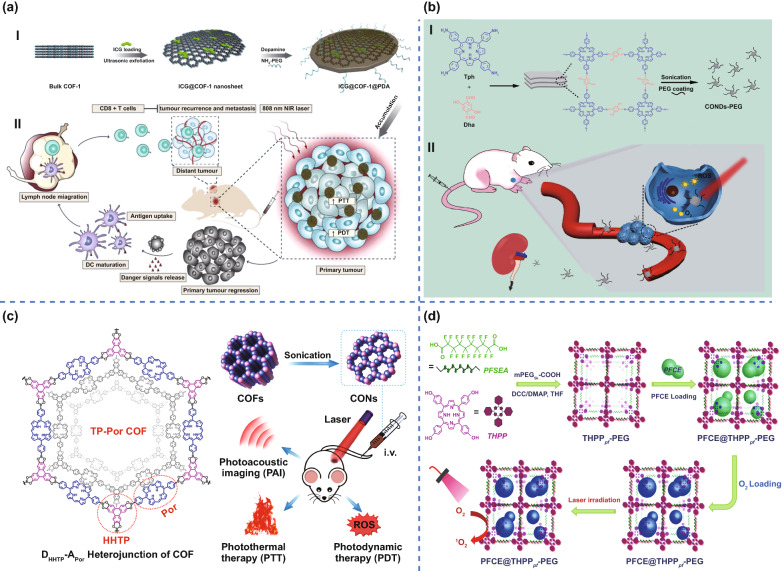
COFs for PDT. **a** I. Fabrication process and II. photoimmunotherapy activity of ICG@COF-1@PDA nanosheets. Adapted with permission [35]. Copyright © 2019 WILEY‐VCH Verlag GmbH & Co. KGaA, Weinheim. **b** Fabrication process of COF nanodots-PEG. Adapted with permission. Adapted with permission [94]. Copyright © 2019 Elsevier Ltd. **c** Schematic illustration of 2D CON fabrication and in vivo cancer therapy. Adapted with permission [90]. Copyright © 2019 American Chemical Society. **d** A scheme illustrating the synthesis route of THPPpf-PEG and the subsequent PFCE loading. Adapted with permission [93]. Copyright © 2018 WILEY‐VCH Verlag GmbH & Co. KGaA, Weinheim

PDT, as a promising treatment method, has attracted considerable research interest [99], which has been applied in clinical cancer therapy. Gan et al. [35] reported a 2D COF nanosheet with loaded photosensitizer indocyanine green (ICG), namely, ICG@COF-1@PDA, which was prepared by loading ICG in COF-1 nanosheet, and subsequently coated with polydopamine (PDA) (Fig. 5aI). After loading, the absorption peak of ICG was obviously redshifted from 779 to 802 nm. Under the 808 nm near infrared (NIR) laser irradiation, the ICG@COF-1@PDA can realize efficient PDT, induce obvious immunogenic cell death (ICD), and elicit antitumor immunity in colorectal cancer, as well as inhibiting untreated distant tumors and metastasis of 4T1 tumor from breast to lung (Fig. 5aII).

As a carrier of PSs, COFs can significantly improve their cellular endocytosis and bioavailability through nanometerization, thereby improving their therapeutic efficacy against cancer. Guan et al. reported two boron-dipyrromethene (BODIPY)-loaded nanoscale COFs (NCOFs), which were synthesized using Schiff-base condensation of the imine-based NCOFs with the organic photosensitizer BODIPY [100]. After loading BODIPY onto NCOFs without photosensitivity, the PDT performance was significantly improved, with low dark toxicity, high phototoxicity, and high tumor treatment efficiency. Similarly, Hu et al. [101] developed a COF–Ag_2_Se nanoparticle, which was successfully prepared at room temperature via a cation exchange process between Ag^+^ and Cu^2+^, using COF and CuSe as templates. Interestingly, COFs can be used as a template to control the size of CuSe or Ag_2_Se, and as an excellent photosensitizer for PDT. The in vitro and in vivo experiment results proved the COF–Ag_2_Se nanoparticle possessed excellent cancer cell-killing effect and in vivo antitumor efficacy.

By rational selection of photosensitive building blocks and π-conjugated structure, COFs with photoactive characteristics can be directly used as PS. Two kinds of COFs with 2D π-conjugated structure were synthesized using 5′,5″″-(1,4-phenylene)bis(([1,1′:3′,1″-terphenyl]-4,4″dicarbaldehyde)) (L-3C) and 4,4′,4″-(1,4-phenylene)bis(([2,2′:6′,2″ terpyridine]-5,5″-dicarbaldehyde)) (L-3 N) as building blocks, which realized PDT for vitro and in vivo cancer therapy [34]. Specifically, the COFs produced a large number of superoxide radicals and hydroxyl radicals under 630 nm red light for a significant killing effect on tumor cells. Zhang et al. prepared COF nanodots (~ 3.46 nm) by a simple liquid exfoliation of a porphyrin-based 2D COF (Fig. 5b) [94]. The experiments on HeLa cells and H22 tumor-bearing mice demonstrated that PEG-coated COF nanodots had high physiological stability and excellent PDT efficiency to inhibit tumor growth. In particular, due to their ultrasmall size COF nanodot-PEGs could be cleared from the body through renal filtration without appreciable in vivo toxicity. This work took advantage of the poor stability of 2D COFs to prepare the COF nanodots with excellent PDT performance, thus providing a new way of thinking about the advantages and disadvantages of the materials.

Wang et al. [90] reported a COF formed by stacking 2D covalent organic nanosheets (CONs) into periodically ordered bicontinuous heterojunction networks and long-range ordered π-columnar structures. The molecular heterostructure of CONs can provide efficient carrier separation and prolong the lifetime of electrons and holes. Meanwhile, electrons can reduce O_2_ to O_2_^−^, which was beneficial to ROS generation through type I PDT mechanism (Fig. 5c). In this experiment, CONs were directly prepared from bulk triphenylene-porphyrin (TP-Por) COFs via liquid ultrasonic exfoliation. Intravenous injection of the resulting CONs into nude mice achieved a significant tumor ablation under 635 nm light irradiation.

PDT often worsens the hypoxia of TME by consuming O_2_ and induces therapy resistance through the action of antioxidant detoxifying enzymes and induction of stress response genes, all of which further reduce the effect of PDT. COF can act as a delivery carrier of oxygen to enhance the PDT effect. Tao et al. [93] reported a unique type of multifunctional fluorinated COF with dual functions of tumor oxygenation and PDT. The authors used photosensitizer meso-5, 10, 15, 20-tetra (4-hydroxylphenyl) porphyrin (THPP), perfluorosebacic acid (PFSEA) and PEG to synthesize COF (THPPpf–PEG) (Fig. 5d). Due to the presence of PFSEA, the obtained THPPpf–PEG had efficient loading of perfluoro-15-crown-5-ether (PFCE) to obtain the PFCE@THPPpf–PEG. Due to the existence of PFCE, the multifunctional PFCE@THPPpf–PEG had effective load of molecular oxygen, and can greatly enhance the tumor oxygenation and PDT effect.

Although research using COFs for PDT is still in its early stages, the excellent photodynamic and sensitization performance of these frameworks have attracted extensive attention. The PDT performance can be adjusted by changing the dimensions, composition, and structure of the COFs. To fully exploit the strengths of COFs for PDT in design and fabrication, two aspects should be fully considered. First, the poor stability of nanomaterials before executing their predetermined task might lead to a limited life span in vivo. Therefore, improving the stability of COFs should be fully considered during material design and fabrication. Second, the photoquenching caused by π–π stacking between COF layers might affect the PDT effect. To improve PDT efficiency, then, the development of COF materials with high electron-transmission efficiency and high electron–hole utilization rate is encouraged.

#### Photothermal Therapy

Photothermal therapy (PTT) utilizes external light (e.g., a laser) to heat tumor cells and induces thermal damage for tissue destruction [102]. PTT has been tried in clinic but is not widely applied, in part because the high-intensity laser might damage normal tissues and cells. Nanoscale photothermal conversion agents (PTAs) that can convert light energy into heat might allow for a lower required light power-intensity while increasing tumor specificity, thus preventing damage to surrounding normal tissues.

During PTT treatment, when tissue temperature rises to higher than 41 °C, it may induce a change in the gene expression pattern and produce a heat-shock protein, so as to discount the cell damage from heat injury. When the temperature rises to 42 °C, irreversible tissue damage will occur; heating the tissue to 42–46 °C for 10 min will lead to cell necrosis. At 46–52 °C, cells die rapidly due to microvascular thrombosis and ischemia. As tissue temperatures > 60 °C, cell death is almost instantaneous owing to protein denaturation and plasma membrane disruption. In general, with the combination of external light irradiation and internal targeted distribution of PTAs, PTT can eliminate tumors while sparing non-malignant tissues. However, this method still has limitations similar to those of other modalities such as chemotherapy and PDT due to the pathological features of cancer. First, tumor tissues are heterogeneous, meaning PTAs are not accessible by some cancer cells. This can result in incomplete tumor ablation with residual cancer cells, which might allow for recurrence and metastasis. Second, the heat-shock response might diminish the therapy response of PTT.

**Fig. 6:**
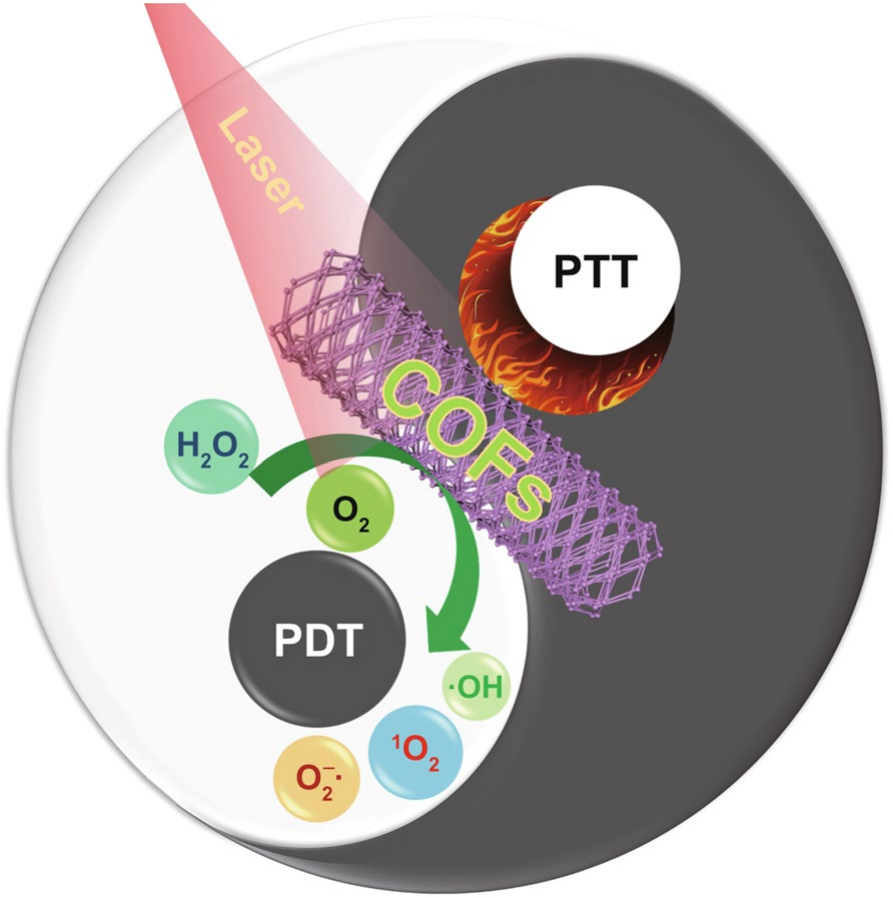
A schematic illustration of phototherapy with COFs

More encouragingly, PDT or PTT can trigger local immunoregulation via immunogenic cell death (ICD), which can synergize the therapy. Moreover, the combination of phototherapy with other therapeutic modalities such as chemotherapy and immunotherapy has the potential to overcome therapy resistance, completely eradicate tumors, and inhibit metastasis. The combination treatment will produce unexpected synergistic effects, as the increased temperature at the tumor site will promote blood flow and oxygen supply. That being said, PDT can interfere with the TME, thereby increasing the tumor’s thermal sensitivity [103]. The optimized collaborative treatment will provide a new direction for improving cancer treatment (Fig. 6).

As a carrier, COFs can load antitumoral drugs, PSs, and PTAs simultaneously. For instance, Dong et al. used a nanoscale COF (~ 140 nm) as a carrier to simultaneously load porphyrinic PS (Por) via covalent graft and naphthalocyanine PTA (VONc) via noncovalent loading [103]. The COFs had an extensive π-conjugated structure, which significantly improved the photostability of the PS and PTA for highly efficient ROS generation and photothermal conversion ability under visible (red LED) and NIR light (808 nm laser) irradiation. Thus deployed, this COF can significantly inhibit MCF-7 breast cancer cell proliferation and systemic metastasis [103]. Chen et al. reported synthesizing porphyrin-based COF nanoparticles (COF-366 NPs) that provided the coordination therapy of PDT and PTT under a single wavelength light (630 nm) [104]. The COF-366 NPs acted as both photosensitizer and photothermal agent, and the conjugated structure broadened the particle’s absorption spectrum. For 4T1 tumor-bearing mice, the treatment accomplished complete inhibition of the tumor growth under 630 nm light irradiation with the combination of PDT and PTT.

#### Other Therapies

The unique characteristics of COFs, such as their extended π-conjugated structure, endow them with additional functions beyond potential phototherapeutic activity. The π–π interaction between the layers of a COF and the existence of heteroatoms can produce different bonding sites with biomolecules in the body, leading to chemical damage. Reasonable usage of this chemical damage can effectively inhibit and treat cancer. Based on this, Bhaumik et al. prepared a novel triazine-based mesoporous 2D COF (TrzCOF) via the solvothermal polycondensation of 1,3,5-tri(4-formylbiphenyl) benzene [Ph_7_(CHO)_3_, TFBPB] with 2,4,6-tris(4-aminophenyl)-1,3,5triazine (TAPT) [36]. In vitro studies showed that the 2D COF had superior anti-tumor activity against colorectal carcinoma HCT-116 cells compared with 5-fluorouracil. They found that cell apoptosis was mediated by p53-guided manner with ROS as an important mediator, which further triggered a train of downstream signal cascades including NOX4 activation, decreased expression of Bcl-2, and augmented caspase 3/caspase 9 activities.

Bhaumik et al. experimented with a porous and biodegradable COF EDTFP-1 (ethylenedianiline-triformyl phloroglucinol), which was synthesized using a Schiff-base condensation reaction of 4,4′-ethylenedianiline and 2,4,6-triformylphloroglucinol [105]. EDTFP-1 exhibited 3D-hexagonal porous structure with average pores of ca. 1.5 nm. The COF had high cytotoxicity to four cancer cells—HCT-116, HepG2, A549, and MIA-Paca2—with significantly lower IC50 for HCT-116 cells. The anti-tumor properties were attributed to ROS generation from polyphenols, p53 phosphorylation induced by EDTFP-1, and EDTFP-1 triggered release of pro-apoptotic protein and suppression of antiapoptotic proteins.

Generally, COFs themselves as therapeutic drugs are still in the early stage of research. However, compared with traditional chemotherapeutic drugs, COFs have some highly advanced functions, such as using bond cooperation for biomolecular interaction and triggering protein expression in vivo.

## COFs for Imaging and Diagnosis

### In vitro Diagnosis

Early diagnosis of cancer plays an increasingly important role in improving cancer therapy. The development of early diagnosis technologies for cancer is of great significance for improving the survival rate of patients. As a kind of organic porous material, COFs have an ordered π-conjugated structure, high porosity, low density, good biocompatibility, and stability; these properties make them excellent carriers for in vitro tumor-detection substances, thus allowing for high sensing sensitivity. At the same time, COFs with high porosity can interact with a greater number of guest molecules, which greatly improves the detection limit. These unique properties of COFs greatly improve the performance of tumor detection.

**Fig. 7:**
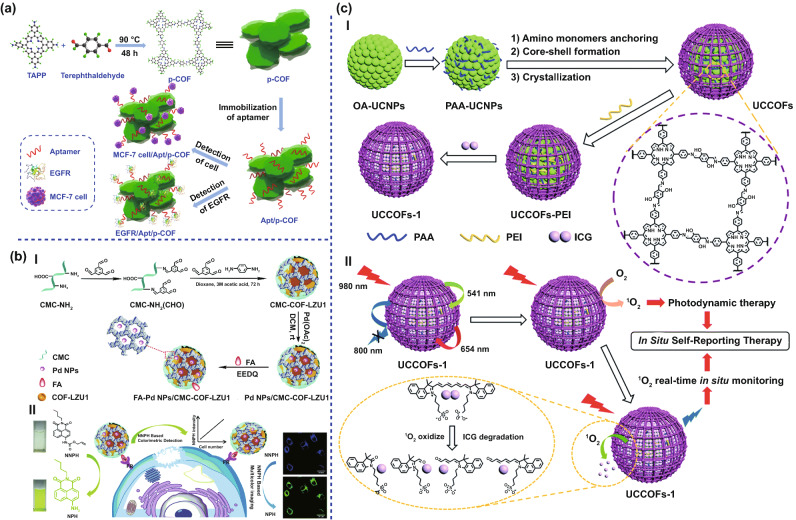
COFs for imaging and diagnosis. **a** Schematic diagram of the p-COF-based aptasensor for detecting EGFR or MCF-7 cells, including (1) preparation of p-COF, (2) immobilization of the aptamer strands, and (3) detection of EGFR or MCF-7 cells. Adapted with permission [106]. Copyright ©2018 Elsevier B.V. All rights reserved. **b** I. Illustration of the synthetic process of FA-Pd NPs/CMC-COF-LZU1. II. Schematic illustration of the dual-function FA-Pd NPs/CMC-COF-LZU1 for cancer cell imaging. Adapted with permission [107]. Copyright © The Royal Society of Chemistry 2020. **c** I. A schematic illustration of the design and synthesis of the upconverting COF nanoplatform UCCOFs-1. II. A schematic illustration of the NIR-excited in situ self-reporting PDT process. Adapted with permission [37]. Copyright © The Royal Society of Chemistry 2020

Based on electrochemical measurement, Liu et al. developed a new aptasensor for immobilizing epidermal growth factor receptor (EGFR)-targeting aptamer strands on a porphyrin COF (p-COF), which can sensitively and selectively bind to EGFR to detect human breast cancer MCF-7 cells (Fig. 7a) [106]. This p-COF-based aptasensor showed a low detection limit (LOD) of 7.54 fg mL^–1^ with a linear detection range of 0.05–100 pg mL^–1^ for EGFR. For MCF-7 cells, the p-COF-based aptasensor showed a LOD of 61 cell mL^–1^ with a linear detection range of 500 × 10^5^ cell mL^–1^. The excellent selectivity and high sensitivity were mainly attributed to the following points: (1) the high conjugation ability and π-conjugated structure of COFs not only increase the interaction between COFs and biomolecules, but also improve the electrochemical activity; (2) the mesoporous channel of COFs (2.06 nm) was conducive to the fixation and loading of aptamers; and (3) the planar structure of 2D COFs allowed more aptamers to be combined with biomolecules. Therefore, the proposed sensor can act as a new biosensing platform for rapid and sensitive detection of cancer biomarkers and cancer cells.

Colorimetric analysis has unique advantages in early diagnosis and point-of-care testing (POCT) of cancer due to its low cost, ease of operation, and high sensitivity. Moreover, COFs are characterized by chemical adjustability, high porosity, ordered structural integrity, and abundant binding sites. Therefore, COFs are becoming an ideal carrier for anchoring various guest molecules. For instance, Wang et al. developed a water-based stable carboxymethyl cellulose-modified COF hydrogel (Pd NPs/CMC-COF-LZU1) based on the principle of colorimetric determination to facilitate the in situ growth of palladium NPs [107]. This composite material enhanced the conversion efficiency of *N*-butyl-4-NHAlloc-1,8-naphthalimide (NNPH) into *N*-butyl-4-amido-1,8naphthalimide (NPH) by improving the activity and stability of Pd NPs, which improved the chromogenic sensitivity (Fig. 7b). Specifically, folic acid (FA)-modified Pd NPs/CMC-COF-LZU1 could target the folate receptor (FR)-positive cancer cells and catalyze NNPH to NPH, which led to color change and multicolor imaging. This detection system can be used to detect cancer cells from serum samples with low detection limit (100 cells per milliliter).

As a porous material, COFs can effectively load fluorescent dyes to minimize the quenching caused by aggregation. At the same time, COFs’ skeleton can not only protect the dyes for good photochemical stability—thus improving the fluorescent properties of the materials—but it also has excellent anti-interference ability, which can function as an excellent fluorescence sensor.

### In vivo Diagnostic Bioimaging

COFs with high surface area and porosity, good electrical conductivity, and special interaction with bioactive molecules have shown great promise in biosensing and bioimaging for cancer diagnosis. For instance, Wang et al. used a lanthanide-doped upconversion nanoparticle (UCNP) core to coat nanoscale COFs (UCCOFs) with various shell thicknesses via core-mediated imine polymerization [37]. The UCCOFs were capable of producing singlet oxygen for PDT, and emit singlet oxygen-correlated fluorescence, allowing real-time and in situ monitoring of the therapeutic process via near-infrared luminescence imaging (Fig. 7c). Specifically, the loaded ICG as a ROS indicator was gradually decomposed by ^1^O_2_. Therefore, the luminescence from UCCOFs-1 at 800 nm was turned on, which reported in real time the position and dose of ^1^O_2_ generation in the body, thus realizing in situ self-reporting PDT.

To increase the high signal-to-noise ratio of imaging and tumor specificity, Liu et al. developed a pH-responsive nanoplatform based on zinc porphyrin COF (ZnCOF), in which the loading rate of zinc porphyrin (ZnPor) was 22.5 wt% [108]. At pH 7.4, the interconnected ZnPor in the assembled state had no fluorescence signal (“off” state). Under the pH-triggered disintegration of ZnCOF in TME (pH = 5.5), the dispersed ZnPor showed obvious fluorescence signal recovery (“on” state). At the same time, the shed bovine serum albumin (BSA)-coated gold nanoparticles further amplified the fluorescence signal through the metal-enhanced fluorescence effect, realizing a ~ 3 times higher fluorescence than the free ZnPor group in vivo. As an excellent carrier, COF can thus interact with imaging molecules to achieve the purpose of imaging. At the same time, the formation of the COF structure can directly integrate the building blocks with fluorescence properties, so the resulting COF can have fluorescence imaging function in a specific environment. Alternately, it can realize the fluorescence activation of inert building blocks after the formation of the COF [109].

To sum up, nanoscale COFs have the following advantages in cancer biosensing and bioimaging: (1) the rich π-conjugation is conducive to sensitive electrochemical signals through π–π stacking interaction with targeting ligands, functional biomolecules, and the like; (2) the optimized particle size for bioavailability can be obtained through COF design; (3) COFs with porous characteristics can fix functional biomolecules such as aptamer inside the material, thereby increasing the adsorption capacity of the probe and its resultant sensing sensitivity; (4) COFs have abundant binding sites and can interact with fluorescent dyes; and (5) COFs constructed from fluorescent molecules can directly have the ability of biological imaging. At the same time, the formation of COF structure can activate the fluorescent activity of the building block.

## Conclusions and Perspectives

In this review, we outline the recent breakthroughs in fabrication and application of COFs for cancer diagnosis and therapy. Given their versatility and physicochemical properties, COFs have showed unique advantages in cancer diagnosis and therapy, especially in drug delivery, phototherapy (including PDT and PTT), biosensing, and bioimaging. With their high surface area and porous nature, COFs also show unique advantages for the delivery of anti-tumor drugs to desired locations. For PDT and PTT, COFs can act not only as carriers of PSs or PTAs due to their porous structure and high surface area, but also as PSs or PTAs by virtue of their high light absorption cross section, π-conjugated structure, and versatile constructions from photoactive building blocks. COFs can effectively avoid the annihilation of PSs caused by π–π stacking and have good thermal stability, high contact area, and light utilization. These properties also help COFs play a role in bioimaging and diagnosis, and imaging-guided therapy.

In spite of these advantages, the study of biomedical applications for COFs is still in the early stages. There are many ongoing challenges and limitations that must be considered and overcome. First, due to the complex structures and arrangements of the pores, it is difficult to precisely control the loading of drugs into and the patterns of release from COFs. In addition, if one more kinds of active molecule (e.g., targeting ligands, drugs, imaging agents) are involved in the system, their location in or on the COFs should be rationally designed. With the abundant active groups acquired during or post-synthesis, the interior capacity and surface area of COFs could be fully utilized via host–guest interaction. Moreover, the structural qualities of COFs, especially their dimensions, have a significant impact on their performance. COFs with different dimensions (2D or 3D) have their respective advantages, and the applications of COFs with other dimensions have rarely been reported. The ingenious and controllable design of COFs’ dimensions is expected to further promote the application of COFs. In terms of possible clinical transformation, the biocompatibility of different nanoscale COFs, especially as regards long-term systemic toxicity, should be a top priority for consideration. Secondly, the large-scale production of nanoscale COFs is still difficult, and further attention is warranted to the study of batch repeatability. Due to the complex molecular arrangement of COFs (e.g., different layer stacking and pore arrangements), the preparation of COFs with stable performance and uniform size distribution in different synthetic batches still poses a great challenge. Finally, possible clinical translation depends on exploring the optimal biocompatible building blocks and achieving the best therapeutic effect through the design of substituents and structures.

Overall, compared with other porous nanomaterials, nanoscale COFs have exhibited unique advantages in the field of cancer diagnosis and therapy. Unlike most of other porous nanomaterials, which act only as inactive carriers for drug delivery and realize other function solely through post-synthetic functionalization, COFs themselves have intrinsic electrical, magnetic, and optical properties that could be applied directly for therapeutic purposes. The synergy of drug delivery and other therapeutic modalities, including phototherapy and immunotherapy, could potentially be realized on a single COF-composed nanosystem, which could resolve many of the challenges of cancer therapy, including difficulty in early diagnosis, therapeutic resistance, recurrence, and metastasis.

## References

[1] Diercks CS, Yaghi OM (2017). The atom, the molecule, and the covalent organic framework. Science.

[2] Hu C, Zhang Z, Liu S, Liu X, Pang M (2019). Monodispersed cuse sensitized covalent organic framework photosensitizer with an enhanced photodynamic and photothermal effect for cancer therapy. ACS Appl. Mater. Interf..

[3] Shi Y, Liu S, Liu Y, Sun C, Chang M (2019). Facile fabrication of nanoscale porphyrinic covalent organic polymers for combined photodynamic and photothermal cancer therapy. ACS Appl. Mater. Interf..

[4] Liu X, Pang H, Liu X, Li Q, Zhang N (2021). Orderly porous covalent organic frameworks-based materials: superior adsorbents for pollutants removal from aqueous solutions. Innovation.

[5] Bhunia S, Deo KA, Gaharwar AK (2020). 2D covalent organic frameworks for biomedical applications. Adv. Funct. Mater..

[6] Chui SS, Lo SM, Charmant JP, Orpen AG, Williams ID (1999). A chemically functionalizable nanoporous material. Science.

[7] Cote AP, Benin AI, Ockwig NW, O'Keeffe M, Matzger AJ (2005). Porous, crystalline, covalent organic frameworks. Science.

[8] Jin E, Li J, Geng K, Jiang Q, Xu H (2018). Designed synthesis of stable light-emitting two-dimensional *sp*(2) carbon-conjugated covalent organic frameworks. Nat. Commun..

[9] Li X, Gao Q, Wang J, Chen Y, Chen ZH (2018). Tuneable near white-emissive two-dimensional covalent organic frameworks. Nat. Commun..

[10] Liu C, Park E, Jin Y, Liu J, Yu Y (2018). Separation of arylenevinylene macrocycles with a surface-confined two-dimensional covalent organic framework. Angew. Chem. Int. Ed..

[11] Jiang C, Tang M, Zhu S, Zhang J, Wu Y (2018). Constructing universal ionic sieves via alignment of two-dimensional covalent organic frameworks (COFs). Angew. Chem. Int. Ed..

[12] Liang RR, Ru-Han A, Xu SQ, Qi QY, Zhao X (2020). Fabricating organic nanotubes through selective disassembly of two-dimensional covalent organic frameworks. J. Am. Chem. Soc..

[13] Lakshmi V, Liu CH, Rajeswara Rao M, Chen Y, Fang Y (2020). A two-dimensional poly(azatriangulene) covalent organic framework with semiconducting and paramagnetic states. J. Am. Chem. Soc..

[14] Li H, Evans AM, Castano I, Strauss MJ, Dichtel WR (2020). Nucleation-elongation dynamics of two-dimensional covalent organic frameworks. J. Am. Chem. Soc..

[15] Hao Q, Li ZJ, Lu C, Sun B, Zhong YW (2019). Oriented two-dimensional covalent organic framework films for near-infrared electrochromic application. J. Am. Chem. Soc..

[16] Colson JW, Woll AR, Mukherjee A, Levendorf MP, Spitler EL (2011). Oriented 2D covalent organic framework thin films on single-layer graphene. Science.

[17] Yu J-T, Chen Z, Sun J, Huang Z-T, Zheng Q-Y (2012). Cyclotricatechylene based porous crystalline material: synthesis and applications in gas storage. J. Mater. Chem..

[18] Han SS, Furukawa H, Yaghi OM, Goddard WA (2008). Covalent organic frameworks as exceptional hydrogen storage materials. J. Am. Chem. Soc..

[19] Furukawa H, Yaghi OM (2009). Storage of hydrogen, methane, and carbon dioxide in highly porous covalent organic frameworks for clean energy applications. J. Am. Chem. Soc..

[20] Lin S, Diercks CS, Zhang Y-B, Kornienko N, Nichols EM (2015). Covalent organic frameworks comprising cobalt porphyrins for catalytic CO_2_ reduction in water. Science.

[21] Stegbauer L, Schwinghammer K, Lotsch BV (2014). A hydrazone-based covalent organic framework for photocatalytic hydrogen production. Chem. Sci..

[22] Ding SY, Gao J, Wang Q, Zhang Y, Song WG (2011). Construction of covalent organic framework for catalysis: Pd/COF-LZU1 in Suzuki-Miyaura coupling reaction. J. Am. Chem. Soc..

[23] Huang N, Ding X, Kim J, Ihee H, Jiang D (2015). A photoresponsive smart covalent organic framework. Angew. Chem. Int. Ed..

[24] Dogru M, Bein T (2014). On the road towards electroactive covalent organic frameworks. Chem. Commun..

[25] Chen L, Furukawa K, Gao J, Nagai A, Nakamura T (2014). Photoelectric covalent organic frameworks: converting open lattices into ordered donor-acceptor heterojunctions. J. Am. Chem. Soc..

[26] Calik M, Auras F, Salonen LM, Bader K, Grill I (2014). Extraction of photogenerated electrons and holes from a covalent organic framework integrated heterojunction. J. Am. Chem. Soc..

[27] F. Zhao, H. Liu, S.D.R. Mathe, A. Dong, J. Zhang, Covalent organic frameworks: from materials design to biomedical application. Nanomaterials **8** (2017). http://doi.org/10.3390/nano8010015 10.3390/nano8010015 PMC5791102 29283423

[28] Guan Q, Zhou LL, Li WY, Li YA, Dong YB (2020). Covalent organic frameworks (COFs) for cancer therapeutics. Chemistry.

[29] Zhang G, Li X, Liao Q, Liu Y, Xi K (2018). Water-dispersible PEG-curcumin/amine-functionalized covalent organic framework nanocomposites as smart carriers for in vivo drug delivery. Nat. Commun..

[30] Mitra S, Sasmal HS, Kundu T, Kandambeth S, Illath K (2017). Targeted drug delivery in covalent organic nanosheets (CONs) via sequential postsynthetic modification. J. Am. Chem. Soc..

[31] Fang Q, Wang J, Gu S, Kaspar RB, Zhuang Z (2015). 3D porous crystalline polyimide covalent organic frameworks for drug delivery. J. Am. Chem. Soc..

[32] Vyas VS, Vishwakarma M, Moudrakovski I, Haase F, Savasci G (2016). Exploiting noncovalent interactions in an imine-based covalent organic framework for quercetin delivery. Adv. Mater..

[33] Wu M-X, Yang Y-W (2017). Applications of covalent organic frameworks (COFs): From gas storage and separation to drug delivery. Chinese Chem. Lett..

[34] Zhang L, Wang S, Zhou Y, Wang C, Zhang XZ (2019). Covalent organic frameworks as favorable constructs for photodynamic therapy. Angew. Chem. Int. Ed..

[35] Gan S, Tong X, Zhang Y, Wu J, Hu Y (2019). Covalent organic framework-supported molecularly dispersed near-infrared dyes boost immunogenic phototherapy against tumors. Adv. Funct. Mater..

[36] Kantidas S, Mishra S, Manna K, Kayal U, Mahapatra S (2018). A new triazine based pi-conjugated mesoporous 2D covalent organic framework: its in vitro anticancer activities. Chem. Commun..

[37] Wang P, Zhou F, Guan K, Wang Y, Fu X (2020). In vivo therapeutic response monitoring by a self-reporting upconverting covalent organic framework nanoplatform. Chem. Sci..

[38] Wang J, Zhao L, Yan B (2020). Indicator displacement assay inside dye-functionalized covalent organic frameworks for ultrasensitive monitoring of sialic acid, an ovarian cancer biomarker. ACS Appl. Mater. Interf..

[39] Yang T, Cui Y, Chen H, Li W (2017). Controllable preparation of two dimensional metal- or covalent organic frameworks for chemical sensing and biosensing. Acta Chim. Sin..

[40] Cui D, Perepichka DF, MacLeod JM, Rosei F (2020). Surface-confined single-layer covalent organic frameworks: design, synthesis and application. Chem. Soc. Rev..

[41] Alahakoon SB, Diwakara SD, Thompson CM, Smaldone RA (2020). Supramolecular design in 2D covalent organic frameworks. Chem. Soc. Rev..

[42] Han X, Yuan C, Hou B, Liu L, Li H (2020). Chiral covalent organic frameworks: design, synthesis and property. Chem. Soc. Rev..

[43] Liang RR, Jiang SY, Ru-Han A, Zhao X (2020). Two-dimensional covalent organic frameworks with hierarchical porosity. Chem. Soc. Rev..

[44] Yu F, Liu W, Li B, Tian D, Zuo JL (2019). Photostimulus-responsive large-area two-dimensional covalent organic framework films. Angew. Chem. Int. Ed..

[45] Zhao Y, Liu H, Wu C, Zhang Z, Pan Q (2019). Fully conjugated two-dimensional sp(2) -carbon covalent organic frameworks as artificial photosystem i with high efficiency. Angew. Chem. Int. Ed..

[46] Zhou D, Tan X, Wu H, Tian L, Li M (2019). Synthesis of C-C bonded two-dimensional conjugated covalent organic framework films by suzuki polymerization on a liquid-liquid interface. Angew. Chem. Int. Ed..

[47] Liang L, Qiu Y, Wang WD, Han J, Luo Y (2020). Non-interpenetrated single-crystal covalent organic frameworks. Angew. Chem. Int. Ed..

[48] Tavakoli E, Kakekhani A, Kaviani S, Tan P, Ghaleni MM (2019). In situ bottom-up synthesis of porphyrin-based covalent organic frameworks. J. Am. Chem. Soc..

[49] Na CG, Ravelli D, Alexanian EJ (2020). Direct decarboxylative functionalization of carboxylic acids via O-H hydrogen atom transfer. J. Am. Chem. Soc..

[50] Dienstmaier JF, Medina DD, Dogru M, Knochel P, Bein T (2012). Isoreticular two-dimensional covalent organic frameworks synthesized by on-surface condensation of diboronic acids. ACS Nano.

[51] Park S, Liao Z, Ibarlucea B, Qi H, Lin HH (2020). Two-dimensional boronate ester covalent organic framework thin films with large single crystalline domains for a neuromorphic memory device. Angew. Chem. Int. Ed..

[52] Chavez AD, Smith BJ, Smith MK, Beaucage PA, Northrop BH (2016). Discrete, hexagonal boronate ester-linked macrocycles related to two-dimensional covalent organic frameworks. Chem. Mater..

[53] Evans AM, Parent LR, Flanders NC, Bisbey RP, Vitaku E (2018). Seeded growth of single-crystal two-dimensional covalent organic frameworks. Science.

[54] Smith BJ, Dichtel WR (2014). Mechanistic studies of two-dimensional covalent organic frameworks rapidly polymerized from initially homogenous conditions. J. Am. Chem. Soc..

[55] Chandra S, Roy Chowdhury D, Addicoat M, Heine T, Paul A (2017). Molecular level control of the capacitance of two-dimensional covalent organic frameworks: role of hydrogen bonding in energy storage materials. Chem. Mater..

[56] Wang P, Zhou F, Zhang C, Yin SY, Teng L (2018). Ultrathin two-dimensional covalent organic framework nanoprobe for interference-resistant two-photon fluorescence bioimaging. Chem. Sci..

[57] Wang M, Ballabio M, Wang M, Lin HH, Biswal BP (2019). Unveiling electronic properties in metal-phthalocyanine-based pyrazine-linked conjugated two-dimensional covalent organic frameworks. J. Am. Chem. Soc..

[58] Kuhn P, Antonietti M, Thomas A (2008). Porous, covalent triazine-based frameworks prepared by ionothermal synthesis. Angew. Chem. Int. Ed..

[59] Jin E, Asada M, Xu Q, Dalapati S, Addicoat MA (2017). Two-dimensional sp(2) carbon-conjugated covalent organic frameworks. Science.

[60] Thomas S, Li H, Dasari RR, Evans AM, Castano I (2019). Design and synthesis of two-dimensional covalent organic frameworks with four-arm cores: prediction of remarkable ambipolar charge-transport properties. Mater. Horiz..

[61] Guan X, Li H, Ma Y, Xue M, Fang Q (2019). Chemically stable polyarylether-based covalent organic frameworks. Nat. Chem..

[62] Li X, Yadav P, Loh KP (2020). Function-oriented synthesis of two-dimensional (2D) covalent organic frameworks-from 3D solids to 2D sheets. Chem. Soc. Rev..

[63] Jakowetz AC, Hinrichsen TF, Ascherl L, Sick T, Calik M (2019). Excited-state dynamics in fully conjugated 2D covalent organic frameworks. J. Am. Chem. Soc..

[64] Meng Z, Stolz RM, Mirica KA (2019). Two-dimensional chemiresistive covalent organic framework with high intrinsic conductivity. J. Am. Chem. Soc..

[65] Sick T, Rotter JM, Reuter S, Kandambeth S, Bach NN (2019). Switching on and off interlayer correlations and porosity in 2D covalent organic frameworks. J. Am. Chem. Soc..

[66] Vitaku E, Dichtel WR (2017). Synthesis of 2D imine-linked covalent organic frameworks through formal transimination reactions. J. Am. Chem. Soc..

[67] Peng Y, Huang Y, Zhu Y, Chen B, Wang L (2017). Ultrathin two-dimensional covalent organic framework nanosheets: preparation and application in highly sensitive and selective DNA detection. J. Am. Chem. Soc..

[68] Wang X, Han X, Zhang J, Wu X, Liu Y (2016). Homochiral 2D porous covalent organic frameworks for heterogeneous asymmetric catalysis. J. Am. Chem. Soc..

[69] Vazquez-Molina DA, Mohammad-Pour GS, Lee C, Logan MW, Duan X (2016). Mechanically Shaped two-dimensional covalent organic frameworks reveal crystallographic alignment and fast li-ion conductivity. J. Am. Chem. Soc..

[70] Bisbey RP, DeBlase CR, Smith BJ, Dichtel WR (2016). Two-dimensional covalent organic framework thin films grown in flow. J. Am. Chem. Soc..

[71] Haug WK, Wolfson ER, Morman BT, Thomas CM, McGrier PL (2020). A nickel-doped dehydrobenzoannulene-based two-dimensional covalent organic framework for the reductive cleavage of inert aryl C-S bonds. J. Am. Chem. Soc..

[72] Ding X, Chen L, Honsho Y, Feng X, Saengsawang O (2011). An n-channel two-dimensional covalent organic framework. J. Am. Chem. Soc..

[73] Xie Z, Wang B, Yang Z, Yang X, Yu X (2019). Stable 2D heteroporous covalent organic frameworks for efficient ionic conduction. Angew. Chem. Int. Ed..

[74] Ma Y, Wang Y, Li H, Guan X, Li B (2020). Three-dimensional chemically stable covalent organic frameworks through hydrophobic engineering. Angew. Chem. Int. Ed..

[75] Feng X, Chen L, Dong Y, Jiang D (2011). Porphyrin-based two-dimensional covalent organic frameworks: synchronized synthetic control of macroscopic structures and pore parameters. Chem. Commun..

[76] Wang Y, Liu Y, Li H, Guan X, Xue M (2020). Three-dimensional mesoporous covalent organic frameworks through steric hindrance engineering. J. Am. Chem. Soc..

[77] Lin G, Ding H, Yuan D, Wang B, Wang C (2016). A pyrene-based, fluorescent three-dimensional covalent organic framework. J. Am. Chem. Soc..

[78] Lanni LM, Tilford RW, Bharathy M, Lavigne JJ (2011). Enhanced hydrolytic stability of self-assembling alkylated two-dimensional covalent organic frameworks. J. Am. Chem. Soc..

[79] Martinez-Abadia M, Stoppiello CT, Strutynski K, Lerma-Berlanga B, Marti-Gastaldo C (2019). A wavy two-dimensional covalent organic framework from core-twisted polycyclic aromatic hydrocarbons. J. Am. Chem. Soc..

[80] Li X, Qiao J, Chee SW, Xu HS, Zhao X (2020). Scalable construction of highly crystalline acylhydrazone two-dimensional covalent organic frameworks via dipole-induced antiparallel stacking. J. Am. Chem. Soc..

[81] Wu X, Han X, Liu Y, Liu Y, Cui Y (2018). Control interlayer stacking and chemical stability of two-dimensional covalent organic frameworks via steric tuning. J. Am. Chem. Soc..

[82] Bi S, Yang C, Zhang W, Xu J, Liu L (2019). Two-dimensional semiconducting covalent organic frameworks via condensation at arylmethyl carbon atoms. Nat. Commun..

[83] Dong J, Li X, Peh SB, Yuan YD, Wang Y (2018). Restriction of molecular rotors in ultrathin two-dimensional covalent organic framework nanosheets for sensing signal amplification. Chem. Mater..

[84] Gao C, Li J, Yin S, Lin G, Ma T (2019). Isostructural three-dimensional covalent organic frameworks. Angew. Chem. Int. Ed..

[85] Lu Q, Ma Y, Li H, Guan X, Yusran Y (2018). Postsynthetic functionalization of three-dimensional covalent organic frameworks for selective extraction of lanthanide ions. Angew. Chem. Int. Ed..

[86] Wang H, Zhu W, Liu J, Dong Z, Liu Z (2018). pH-responsive nanoscale covalent organic polymers as a biodegradable drug carrier for combined photodynamic chemotherapy of cancer. ACS Appl. Mater. Interf..

[87] Wang K, Zhang Z, Lin L, Hao K, Chen J (2019). Cyanine-assisted exfoliation of covalent organic frameworks in nanocomposites for highly efficient chemo-photothermal tumor therapy. ACS Appl. Mater. Interf..

[88] Wang SB, Chen ZX, Gao F, Zhang C, Zou MZ (2020). Remodeling extracellular matrix based on functional covalent organic framework to enhance tumor photodynamic therapy. Biomaterials.

[89] Akyuz L (2020). An imine based COF as a smart carrier for targeted drug delivery: From synthesis to computational studies. Micropor. Mesopor. Mater..

[90] Wang K, Zhang Z, Lin L, Chen J, Hao K (2019). Covalent organic nanosheets integrated heterojunction with two strategies to overcome hypoxic-tumor photodynamic therapy. Chem. Mater..

[91] Dai H, Shen Q, Shao J, Wang W, Gao F (2021). Small molecular NIR-II fluorophores for cancer phototheranostics. Innovation.

[92] Tan H, Kong P, Zhang R, Gao M, Liu M (2021). Controllable generation of reactive oxygen species on cyano-group-modified carbon nitride for selective epoxidation of styrene. Innovation.

[93] Tao D, Feng L, Chao Y, Liang C, Song X (2018). Covalent organic polymers based on fluorinated porphyrin as oxygen nanoshuttles for tumor hypoxia relief and enhanced photodynamic therapy. Adv. Funct. Mater..

[94] Zhang Y, Zhang L, Wang Z, Wang F, Kang L (2019). Renal-clearable ultrasmall covalent organic framework nanodots as photodynamic agents for effective cancer therapy. Biomaterials.

[95] Wang H, Zhu W, Feng L, Chen Q, Chao Y (2018). Nanoscale covalent organic polymers as a biodegradable nanomedicine for chemotherapy-enhanced photodynamic therapy of cancer. Nano Res..

[96] Qian Y, Li D, Han Y, Jiang HL (2020). Photocatalytic molecular oxygen activation by regulating excitonic effects in covalent organic frameworks. J. Am. Chem. Soc..

[97] Liu S, Yang J, Guo R, Deng L, Dong A (2020). Facile fabrication of redox-responsive covalent organic framework nanocarriers for efficiently loading and delivering doxorubicin. Macromol. Rapid Commun..

[98] Y. Ding, Y. Dai, M. Wu, L. Li, Glutathione-mediated nanomedicines for cancer diagnosis and therapy. Chem. Eng. J. **128880** (2021). 10.1016/j.cej.2021.128880

[99] Zhao Y, Dai W, Peng Y, Niu Z, Sun Q (2020). A corrole-based covalent organic framework featuring desymmetrized topology. Angew. Chem. Int. Ed..

[100] Guan Q, Fu DD, Li YA, Kong XM, Wei ZY (2019). BODIPY-decorated nanoscale covalent organic frameworks for photodynamic therapy. iScience.

[101] Hu C, Cai L, Liu S, Pang M (2019). Integration of a highly monodisperse covalent organic framework photosensitizer with cation exchange synthesized Ag_2_Se nanoparticles for enhanced phototherapy. Chem. Commun..

[102] Li X, Lovell JF, Yoon J, Chen X (2020). Clinical development and potential of photothermal and photodynamic therapies for cancer. Nat. Rev. Clin. Oncol..

[103] Guan Q, Zhou LL, Li YA, Li WY, Wang S (2019). Nanoscale covalent organic framework for combinatorial antitumor photodynamic and photothermal therapy. ACS Nano.

[104] Wang D, Zhang Z, Lin L, Liu F, Wang Y (2019). Porphyrin-based covalent organic framework nanoparticles for photoacoustic imaging-guided photodynamic and photothermal combination cancer therapy. Biomaterials.

[105] Bhanja P, Mishra S, Manna K, Mallick A, Das Saha K (2017). Covalent organic framework material bearing phloroglucinol building units as a potent anticancer agent. ACS Appl. Mater. Interf..

[106] Yan X, Song Y, Liu J, Zhou N, Zhang C (2019). Two-dimensional porphyrin-based covalent organic framework: A novel platform for sensitive epidermal growth factor receptor and living cancer cell detection. Biosens. Bioelectron..

[107] Sun P, Hai J, Sun S, Lu S, Liu S (2020). Aqueous stable Pd nanoparticles/covalent organic framework nanocomposite: an efficient nanoenzyme for colorimetric detection and multicolor imaging of cancer cells. Nanoscale.

[108] Liu Y, Zhang Y, Li X, Gao X, Niu X (2019). Fluorescence-enhanced covalent organic framework nanosystem for tumor imaging and photothermal therapy. Nanoscale.

[109] Zeng JY, Wang XS, Xie BR, Li MJ, Zhang XZ (2020). Covalent organic framework for improving near-infrared light induced fluorescence imaging through two-photon induction. Angew. Chem. Int. Ed..

